# On the Electrochemical Migration Mechanism of Gold in Electronics—Less Reliable Than Expected?

**DOI:** 10.3390/ma14185237

**Published:** 2021-09-12

**Authors:** Bálint Medgyes, Ali Gharaibeh, Dániel Rigler, Gábor Harsányi

**Affiliations:** Department of Electronics Technology, Faculty of Electrical Engineering and Informatics, Budapest University of Technology and Economics, 1111 Budapest, Hungary; aligharaibeh@ett.bme.hu (A.G.); rigler@ett.bme.hu (D.R.); harsanyi@ett.bme.hu (G.H.)

**Keywords:** electrochemical migration, electronics, gold, anodic dissolution

## Abstract

Electrochemical migration (ECM) forming dendritic short circuits is a major reliability limiting factor in microcircuits. Gold, which is a noble metal, has been regarded as a metallization that can withstand corrosion and also ECM, therefore its application in high-reliability metallization and surface finishing systems became widespread although it has a relatively high and fluctuating price. Gold electrochemical short circuits have been found only in the case of halogen (e.g., chloride containing) contaminants that can initiate the anodic dissolution of gold via complex ion formation. The experimental results of the study demonstrate that gold can form dendritic shorts even without the presence of halogen contaminants, therefore the direct anodic dissolution of gold must also be supposed. This could also be a serious reliability influencing factor even when applying gold metallization systems and must be taken into consideration. The theoretical background of the classical (contaminant-free) model of gold is also discussed in the paper.

## 1. Introduction

Various electrochemical corrosion-related failures [1,2] have received increased attention due to the current miniaturization trend in terms of electronics. One of these corrosion-related failure mechanisms is ionic or electrochemical migration (ECM) [3,4]. The first electrochemical model of ECM was reported regarding Ag migration [5]. In the so-called classical ECM model, dendrites grow from the cathode towards the anode in an aqueous electrolyte without any contaminants. The migrated electrical shorts appear at random locations on the surface and emerge mainly under extraordinary conditions (i.e., fluctuating temperature and moisture). Later, it was discovered that several other metals can also show signs of ionic migration and can form dendrites, such as copper, lead, tin, nickel, gold, etc. However, contradictory statements can be read in the literature about the ECM behavior of gold.

On one hand, the ECM susceptibility of Au was first detected on the surface finishes of printed circuit boards (PCBs). On the other hand, numerous other investigations have also found ECM processes of gold on ceramic thick film circuits as well as in ceramic packages [6,7,8,9,10,11,12,13,14,15]. The reports described that the ionic surface contaminants (mainly halogens such as chloride) can act a significant part in the case of gold ECM, while in chloride ion-free cases, migration was not detected. According to the early publications, essentially Cl_2_ gas evolution and/or tetra-chloro-gold complex ion formation was supposed to occur on the anode [10,12]:

Au + 4Cl^−^ → AuCl^−^_4_ + 3e^−^ (1)

However, the formed negatively charged complex ion in Equation (1) leads to a contradiction. The main problem of this model is that the electrochemical process according to Equation (1) leads to the formation of anions, therefore their migration toward the cathode can hardly be imagined. On the other hand, tetra-chloro-gold complex ions might be unstable and further chemical reactions had to be assumed. The main forms of gold in solutions are complex ions of the type [16]:

[Au(OH)_x_Cl_y_]^(x + y − 4)−^, where (x + y < 4) (2)

Among these complexes, cations may also be present (for example, when x = y = 1), the migration of which toward the cathode is possible.

According to another theoretical explanation [17], the following chemical “chain reaction” is also possible in acidic media, which are present in the anode region (since the deposition of OH^−^ ions results in an enrichment of H^+^ ions there) [16,17]:

AuCl^−^_4_ + H^+^ → H[AuCl_4_] → HCl + AuCl_3_ → H^+^ + Au^3+^ + 4Cl^−^ (3)

The resulting Au^3+^ ions are positive metal ions that can migrate toward the cathode and can form dendrites similar to the classical model. Generally, the process of contaminant-induced migration can be summarized in the following steps [5,16]:

1. Primary negative complex ion formation by anodic corrosion induced by halogen contaminants (see Equation (1)).
2. A multi-step chemical process resulting in metal ions or secondary complex cations (Equations (2) and (3)).
3. Cation migration through the electrolyte under the electrical field toward the cathode.
4. Electrochemical deposition at the cathode forming metallic dendrites.

It should also be noted that some researchers published reports about the ECM of gold without any contaminants. According to Noh et al., gold can also follow the classical ECM model [18,19]. Accordingly, it is certain that gold has an ability for migration, but the migration mechanisms of gold and its circumstances need further clarifications.

In many cases, complex metallization systems (multi-layer or compound structures) are investigated. Sputtered or evaporated pure gold thin films cannot be realized on bare substrates because of the pore adhesion of the film, therefore, underlying chromium, nickel, or titanium–platinum/palladium film is always present that can strongly influence the electrode potential of the film and thus the electrochemical processes on the surface [10,15]. Gold finishes on printed wiring boards are generally prepared on copper with a nickel diffusion barrier interface (such as electroless nickel, immersion gold (ENIG)) and may contain alloying compounds such as nickel, or other chemical species like phosphor [18,19,20,21,22]. To avoid the effect of metallic compounds other than gold, thick film technology with pure gold metallization was used for sample preparation. Thick films generally also contain glass frit as an inorganic binder material that can also influence the migration processes, but these glasses do not provide ionic compounds for the migration itself. The situation is worse with oxide frits that may show “virtual migration”, e.g., in the case of copper oxide, but this can be distinguished from gold processes [5,8,23,24]. Therefore, in this study, the ECM behavior of Au was investigated on cleaned thick film samples (test boards) with conductive lines made of glass fritted gold paste. The experiment was performed using water drop (WD) tests [25].

## 2. Experimental

**Figure 1:**
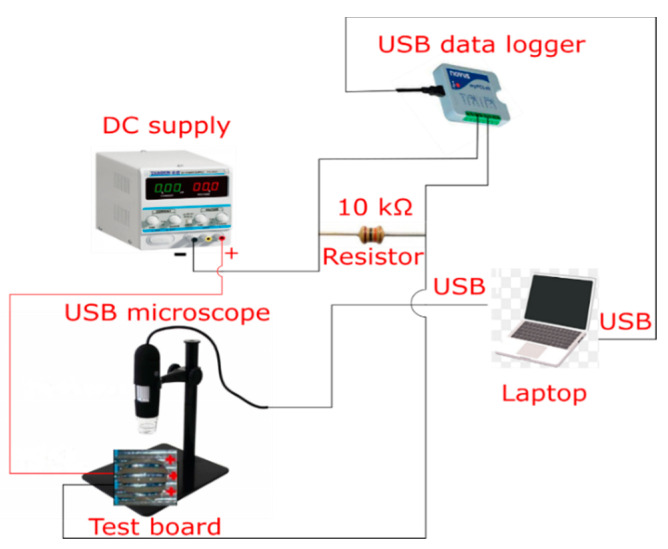
Schematic of the measuring platform for ECM WD tests.

The samples were fabricated by using classical thick film technology using 96% alumina ceramic substrates. A glass bonded pure gold thick film metallization paste was applied by screen printing and it was fired at 850 °C. The thickness of the film was around 12 μm. A simple structure with two parallel strips with a gap of 0.2 mm was used for the ECM tests. The test setup (Figure 1) was able to provide the in situ observation of the dendrite formation with the use of an optical inspection system while recording real-time voltage data on a series resistor [26]. According to the voltage data, dendrite growth processes were followed, and time-to-failure values were determined.

**Figure 2:**
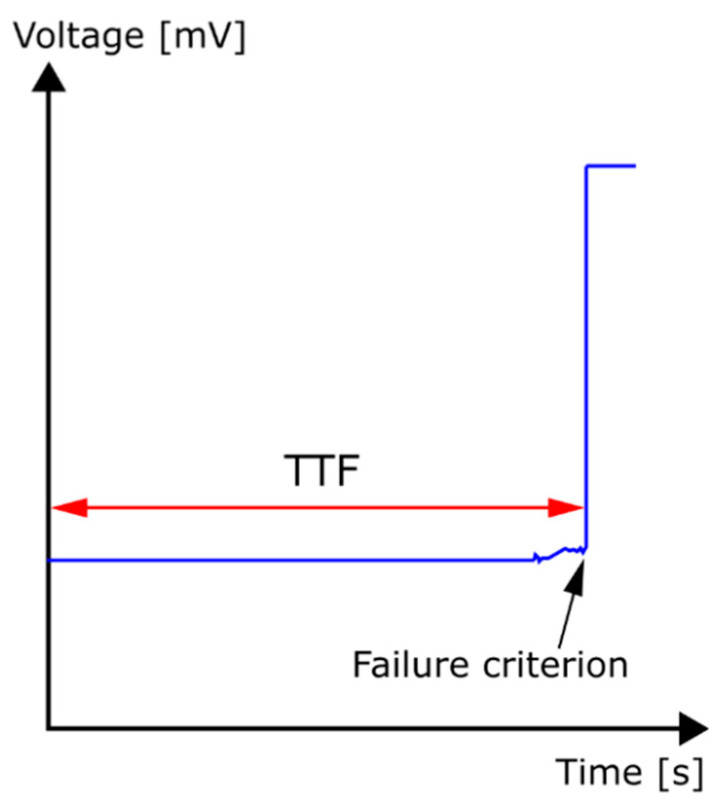
Example of time-to-failure value determination with voltage jump, as failure criterion.

The test solution used for the water drop (WD) tests was double-distilled water (18.2 MΩcm) to minimize the effect of contaminants and to check the ECM suitability of gold to the classical model. Before each test, the surface of the test board was washed out with deionized (DI) water and was also cleaned with isopropyl alcohol (IPA). In each experiment, a droplet of 10 µL of double-distilled water was placed onto the test surface. Between the electrodes, 1, 3, 5, and 10 VDC biases were used. Time-to-failure (short-circuit) measurement from voltage diagrams was carried out based on the “first-jump” phenomenon. Therefore, the failure criterion was the first appearance of a significant jump in the voltage signal (Figure 2). All ECM tests were repeated more than ten times to reach adequate reproducibility.

After the tests, photographs were taken from the surface of the test boards using optical microscopy. Scanning electron microscopy and energy-dispersive X-ray spectroscopy (SEM-EDS, FEI Inspect S50 and Bruker Quanta EDX) methods were also used to investigate the composition of the dendrites and precipitates on the boards.

## 3. Results of the WD and SEM-EDS Investigations

**Figure 3:**
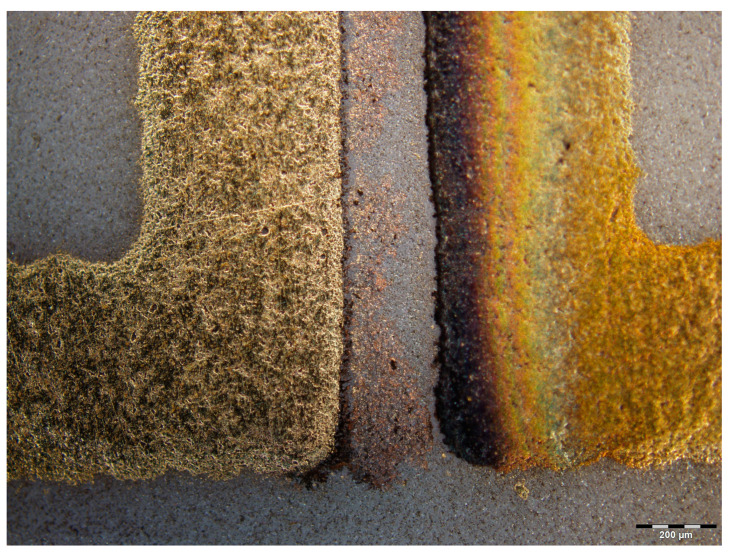
Gold deposits and dendrites on the samples after WD tests performed with 5 V bias.

**Figure 4:**
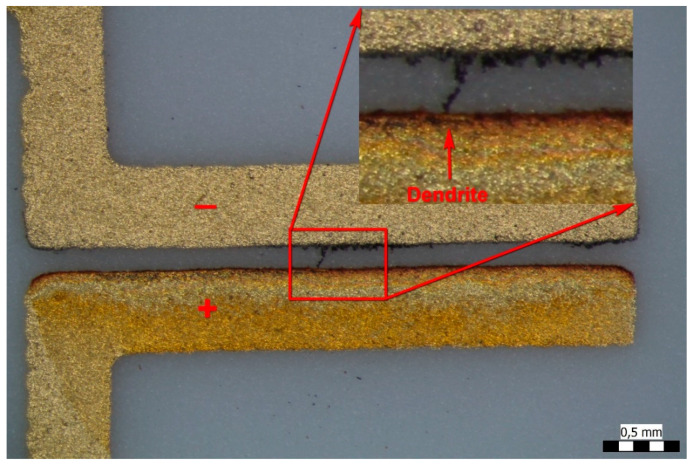
Gold dendrites after a WD test with an applied 10 V bias potential.

In the cases of 1 V and 3 V bias potential, no dendritic growth could be observed in the experiments, but oxidization of the gold surfaces occurred. In the cases of the 5 and 10 V experiments (see Figure 3 and Figure 4, respectively), both oxidization and dendritic growth could be observed, as shown in the optical photographs.

**Figure 5:**
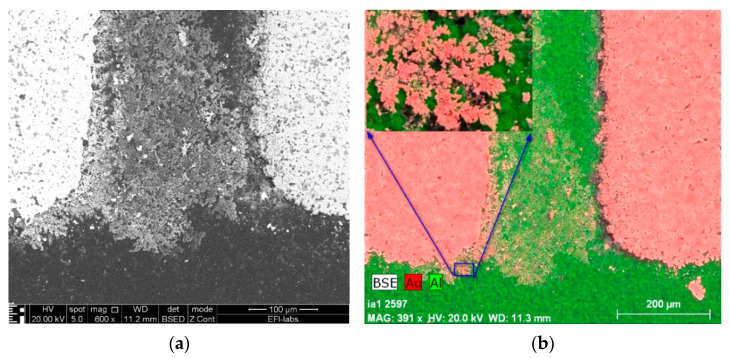
SEM micrographs of the structure shown in Figure 3: (**a**) backscattered electron micrograph; (**b**) backscattered electron micrograph with a color overlay representing the elemental distribution by X-ray mapping (Au red, Al green), (with 5 V bias).

**Figure 6:**
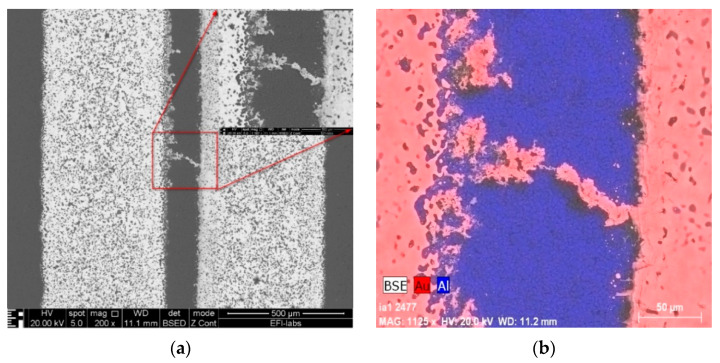
SEM micrographs of the structure shown in Figure 4: (**a**) backscattered electron micrograph; (**b**) backscattered electron micrograph with a color overlay of the elemental distribution by X-ray mapping (Au red, Al blue), (with 10 V bias).

It can be seen that when applying 5 V (Figure 5), the dendrites and the gold deposition cover a larger area in comparison with the sample prepared with 10 V (Figure 6), where the dendritic growth is much more concentrated. One reason for this could be the difference in the speed of the dendrite growth between both samples: the slower the process, the larger its extension before the short circuit occurrence. Furthermore, the oxidation seems to be stronger in the slower 5 V process thanks to the longer duration of the anodic electrochemical process during the WD test.

SEM-EDS methods were also applied to analyze the composition of the dendrites and residues on the boards. Surface morphology is shown in the secondary electron (SE) images, while backscattered electron (BSE) images give elemental contrast according to the atomic number of the elements. Elemental X-ray mapping can be added to SEM pictures where the different colors represent the distribution of the elements. Energy-dispersive X-ray spectra can be used for qualitative and quantitative chemical analysis (See Figure 5 and Figure 6).

**Figure 7:**
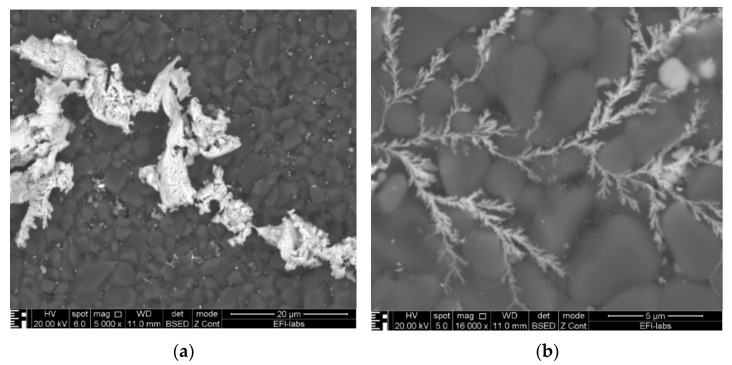
Close-up SEM micrographs of the dendrite structure: (**a**) rough and (**b**) very fine line Scheme 10 V bias.

**Figure 8:**
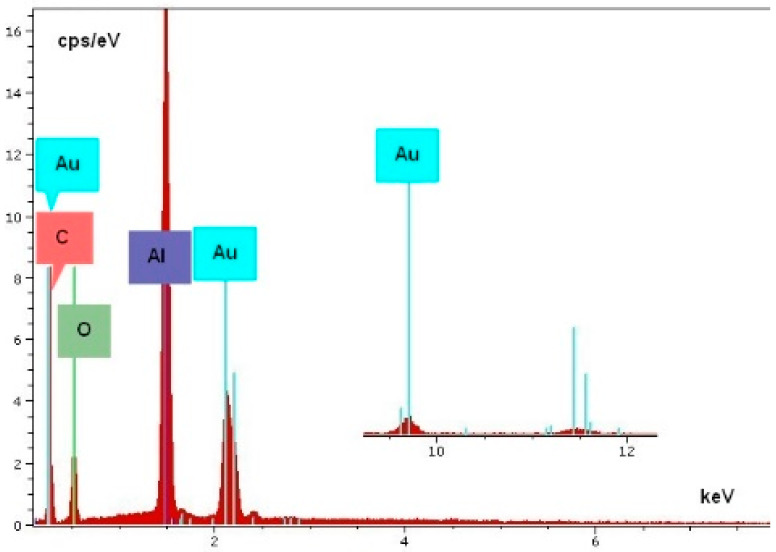
Typical EDS spectrum of the thin dendrite structures on the substrate.

**Figure 9:**
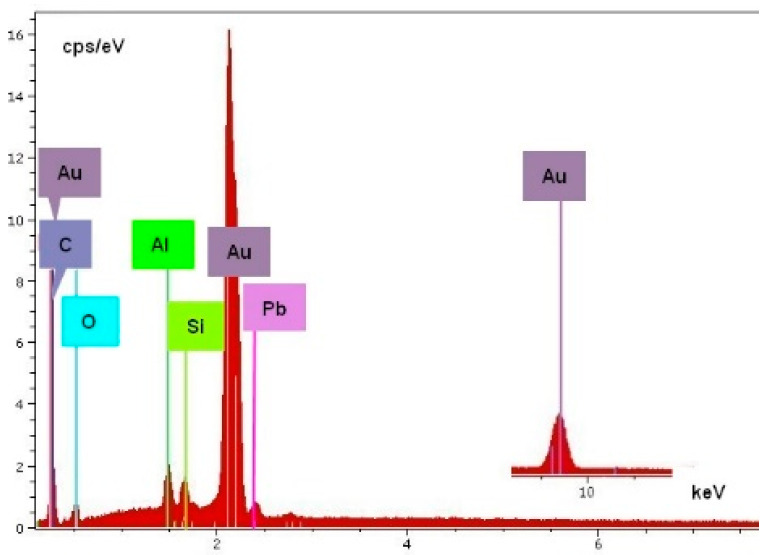
Typical EDS spectrum of the conductive layer.

Figure 7 indicates, with the spectrum in Figure 8, that the material of the dendrite is pure gold. The spectrum in Figure 9 came directly from the conductive layer. The Al line originates from the substrate, while the Si and Pb lines indicate the presence of classical frit material inside the conductive layer: lead-borosilicate glass (PbO/B2O3/SiO2). In the experiments, a retro-type frit bonded gold paste was used to prevent the migration of reduced oxide compounds which is typical for oxide bonded pastes [5,24]. A typical spectrum of the dendrites is shown in Figure 8. The samples were covered by a thin carbon layer to avoid electrical charging during the analysis. It can be seen that gold and aluminum lines are dominating the spectrum. Aluminum and oxygen are present because the substrate is alumina ceramic, and carbon is the cover layer. No other elements could be detected, not even any halogen contaminants (such as chlorides).

## 4. Discussion

Electrochemical corrosion behavior of gold has been studied by chemical experts for many years [27,28,29,30,31]. The investigated circumstances are, however, very different from those that are typically present in microcircuits. In the latter case, no real electrolyte is available: the dew is formed by humidity deposition that results in practically clean water and only the dissolved surface contaminants may form an electrolyte. Often, processes within the very thin high-purity water film should be supposed, therefore the WD tests were performed with double-distilled water. Low voltage bias in microelectronics means a level under 10 V, which is, however, very high in comparison with the typical standard electrode potentials and polarization voltages used in electro-analysis.

The experiments and the analyses have demonstrated here that pure gold dendrites were formed during the WD tests between the gold electrodes. No contaminants were found on the samples, neither on the substrate nor on the conductive layer and dendrites. Therefore, it must be supposed that gold can also follow the classical model of ECM without complex ion formation:

1. Au ion formation by anodic corrosion:

Au → Au^3+^ + 3e^−^ (4)

2. Cation migration through the electrolyte under an electrical field toward the cathode.
3. Electrochemical deposition at the cathode forming metallic dendrites:

Au^3+^ + 3e^−^ → Au (5)

The gold ECM process was found between the electrodes only in the case of relatively high bias: 5–10 Volts. In the case of 1 and 3 V, no ECM process was found. This can also resolve the contradictory statements of the literature in connection with the migration of pure gold—it may start only above a threshold polarization voltage level—the value of which may vary with many other characteristics (such as geometry, substrate material, etc.). According to the Pourbaix diagram of gold [32], it is theoretically possible that in the case of “higher” polarizing potential ranges (outside of the water dissociation zone), Au^3+^ ions can be formed in pure water. This confirms the abovementioned ECM model of gold.

## 5. Conclusions

Electrochemical migration (ECM) forming dendritic short circuits is a major reliability limiting factor in the electronics industry. Gold is a noble metal which has been regarded as a metallization/surface finish that can withstand corrosion and also ECM for many years. Gold electrochemical short circuits have been found only in the case of halogen-rich (e.g., chloride) contaminants to date. A WD test was performed with double-distilled water on pure gold thick film conductors. The EDS analysis of the samples demonstrated that gold can form dendritic shorts even without the presence of a halogen-rich contaminant, but this is electrical potential dependent. Therefore, the direct anodic dissolution of gold must also be supposed. On one hand, this means that gold can follow the so-called classical (contaminant-free) model next to the known contaminant-induced ECM model. On the other hand, this also means a serious reliability influencing factor when applying gold metallization systems and must be taken into consideration in the material design aspects of microcircuits.

## Data Availability

The data presented in this study are available on request from the corresponding author. The data are not publicly available due to confidentiality agreement.

## References

[1] Verdingovas V., Jellesen M.S., Ambat R. (2015). Solder Flux Residues and Humidity-Related Failures in Electronics: Relative Effects of Weak Organic Acids Used in No-Clean Flux Systems. J. Electron. Mater..

[2] Pour Z.S., Ghaemy M., Bordbar S., Karimi-Maleh H. (2018). Effects of surface treatment of TiO_2_ nanoparticles on the adhesion and anticorrosion properties of the epoxy coating on mild steel using electrochemical technique. Prog. Org. Coat..

[3] Harsányi G. (1999). Copper may destroy chip-level reliability: Handle with care—Mechanism and conditions for copper migrated resistive short formation. IEEE Electron. Device Lett..

[4] Zhong X., Yu S., Chen L., Hu J., Zhang Z. (2017). Test methods for electrochemical migration: A review. J. Mater. Sci. Mater. Electron..

[5] Harsanyi G. (1995). Electrochemical Processes Resulting in Migrated Short Failures in Microcircuits. IEEE Trans. Compon. Packag. Manuf. Technol. Part A.

[6] Zhan S., Azarian M.H., Pecht M. (2008). Reliability of printed circuit boards processed using no-clean flux technology in temperature-humidity-bias conditions. IEEE Trans. Device Mater. Reliab..

[7] Warren G.W., Wynblatt P., Zamanzadeh M. (1989). The Role of Electrochemical Migration and Moisture Adsorption on the Reliability of Metallized Ceramic Substrates. J. Electron. Mater..

[8] Ripka G., Harsanyi G. (1985). Electrochemical Migration in Thick-Film Ic-S. Electrocompon. Sci. Technol..

[9] DerMarderosian A. The Electrochemical Migration of Metals. Proceedings of the 11th International Microelectronics Symposium.

[10] Sbar N.L. (1976). Bias-Humidity Performance of Encapsulated and Unencapsulated Ti-Pd-Au Thin-Film Conductors in an Environment Contaminated with Cl_2_. IEEE Trans. Parts Hybrids Packag..

[11] Christou A., Griffith J.R., Wilkins B.R. (1979). Reliability of Hybrid Encapsulation Based on Fluorinated Polymeric Materials. IEEE Trans. Electron Devices.

[12] Grunthaner F., Griswold T.W., Clendening P.J. Migratory Gold Resistive Shorts: Chemical Aspects of a Failure Mechanism. Proceedings of the 13th IEEE International Reliability Physics Symposium.

[13] Wright J.C. Reliability Improvements of Plastic Semiconductors using Gold Metallization. Proceedings of the 11th IEEE International Reliability Physics Symposium.

[14] Shumka A., Piety R.R. Migrated-Gold Resistive Shorts in Microcircuits. Proceedings of the 13th IEEE International Reliability Physics Symposium.

[15] Hakim E.B., Shappiro I.R. (1975). Failure Mechanism in Gold Metalized Sealed Junction Devices. Solid State Technol..

[16] Harsányi G. (1999). Irregular effect of chloride impurities on migration failure reliability: Contradictions or understandable?. Microelectron. Reliab..

[17] Gaur J.N., Schmid G.M. (1970). Electrochemical behavior of gold in acidic chloride solutions. J. Electroanal. Chem..

[18] Noh B.I., Lee J.B., Jung S.B. (2008). Effect of surface finish material on printed circuit board for electrochemical migration. Microelectron. Reliab..

[19] Noh B.I., Yoon J.W., Hong W.S., Jung S.B. (2009). Evaluation of electrochemical migration on flexible printed circuit boards with different surface finishes. J. Electron. Mater..

[20] Medgyes B. (2017). Electrochemical migration of Ni and ENIG surface finish during Environmental test contaminated by NaCl. J. Mater. Sci. Mater. Electron..

[21] Hong W.S., Oh C. (2019). Lifetime Prediction of Electrochemical Ion Migration with Various Surface Finishes of Printed Circuit Boards. J. Electron. Mater..

[22] Yi P., Xiao K., Ding K., Dong C., Li X. (2017). Electrochemical migration behavior of copper-clad laminate and electroless nickel/immersion gold printed circuit boards under thin electrolyte layers. Materials.

[23] Harsányi G. (1996). Material design aspects of high reliability, high density interconnections. Mater. Chem. Phys..

[24] Harsányi G. (1992). Dendritic Growth from Dielectric Constituents in Thick Film ICs. Microelectron. Int..

[25] Matthew L.C., Rath D.L. The waterdrop test—Highly accelerated migration testing. Proceedings of the Materials Development in Microelectronic Packaging Conference.

[26] Medgyes B., Illés B., Berényi R., Harsányi G. (2011). In situ optical inspection of electrochemical migration during THB tests. J. Mater. Sci. Mater. Electron..

[27] Burke L.D., Nugent P.F. (1997). The electrochemistry of gold: I. The redox behaviour of the metal in aqueous media. Gold Bull..

[28] Nicol M.J. (1980). The anodic behaviour of gold—Part I—Oxidation in acidic solutions. Gold Bull..

[29] Nicol M.J. (1980). The anodic behaviour of gold—Part II—Oxidation in alkaline solutions. Gold Bull..

[30] Oesch U., Janata J. (1983). Electrochemical study of gold electrodes with anodic oxide films—I. Formation and reduction behaviour of anodic oxides on gold. Electrochim. Acta.

[31] Massey A.G., Thompson N.R., Johnson B.F.G., Davis R. (1975). The Chemistry of Copper, Silver and Gold.

[32] Van Muylder J., Pourbaix M. (1974). Atlas of Electrochemical Equilibria in Aqueous Solutions.

